# Supplementary material for: Anomalous electronic transport in high-mobility Corbino rings
Source: Nat Commun. 2023 Jul 3;14:3906. doi: 10.1038/s41467-023-39526-x (PMC10318031; doi:10.1038/s41467-023-39526-x)
Supplement: Supplementary file 1 — Supplementary Information [file 41467_2023_39526_MOESM1_ESM.pdf]

Supplementary Material

1 Heterostructure

A sketch of the wafer heterostructure used to fabricate CBM301, VdP301 and CBM302, VdP302 is shown in Fig.S1. The main components forming the heterostructure grown on an undoped GaAs subtrate are: a buffer/spacer, the setback defining the position of the dopants, the quantum well width, and the spacer/capping layer. The main differences between the 301 and 302 heterostructures are: the electron density (3.6×10^{11} cm^{-2} for CBM301 and 1.7×10^{11} cm^{-2} for CBM302); the quantum well width (30 nm for CBM301 and 40 nm for CBM302); the setback distance for the dopants (∼80 nm for CBM301 and ∼160 nm for CBM302).

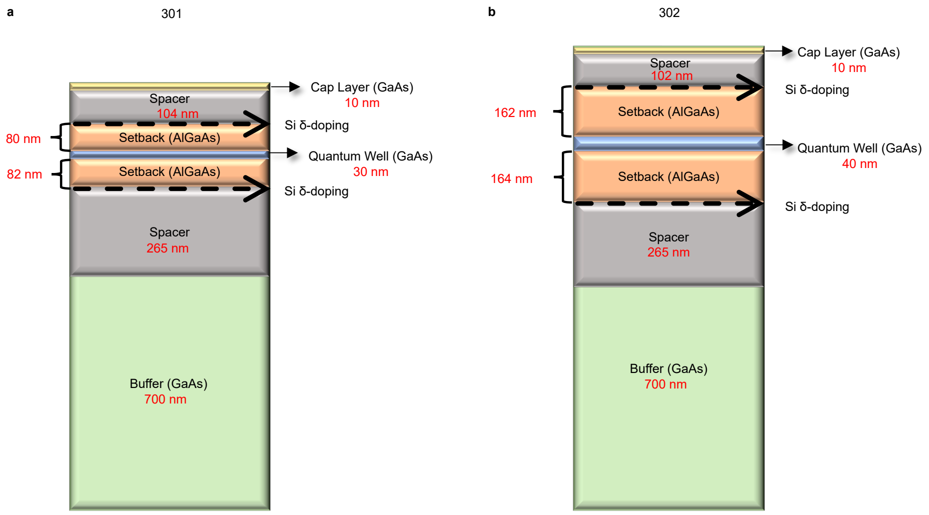

Figure S1: Heterostructure of (a) 301 (CBM301, VdP301) and (b) 302 (CBM302, VdP302). The main heterostructure components and their thickness are shown.

2 Electronic transport measurement circuit

In the main text of the manuscript, Fig.2 shows the conductance of Corbino devices measured with two different electrical setups, and these are discussed below.

2.1 Experimental setup A

The experimental setup A shown in Fig.S2 consists of a SR830 lock-in amplifier and a resistor with a high resistance. An output voltage of 200 mV is passed through a 10 $M\Omega$ resistor connected in series with the Corbino device. This configuration allows us to apply a constant current of 20 nA

to the outermost contact of the Corbino while keeping the inner contact grounded. The four-point resistance is calculated from the voltage drop measured across the inner two rings of the Corbino samples.

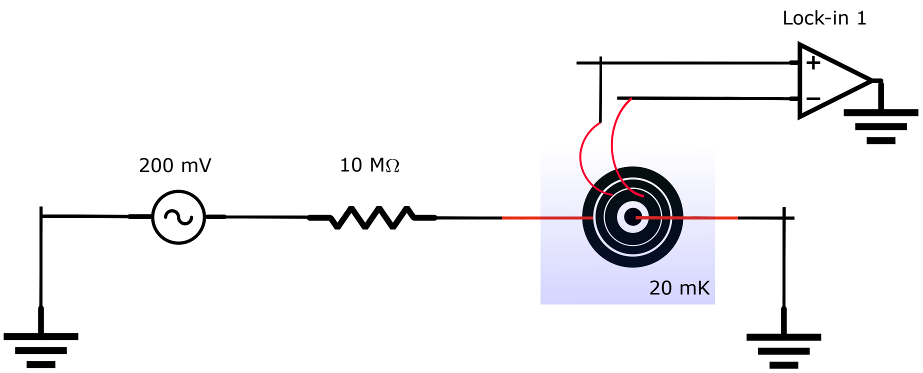

Figure S2: Setup A circuit used to determine the four point resistance of the Corbino sample with a fixed current of 20 nA.

2.2 Experimental setup B

In the Corbino geometry, the sample has a large magneto-resistance in non-zero magnetic fields, which makes the previous experimental setup not ideal for this case. Experimental setup B was used to measure the conductance while sweeping the magnetic field in order to extract the electron density from the Shubnikov de-Haas (SdH) oscillations. In this configuration, an output voltage of 100 mV was applied to a voltage divider consisting of 100 $k\Omega$ $\|$ 100 Ω, connected in series with the Corbino. The current through the sample is calculated from the measured voltage drop across a 1 $k\Omega$ resistor connected in series with the Corbino. For both experimental setups A and B, a voltage pre-amp with the gain of 100 was used at the output end.

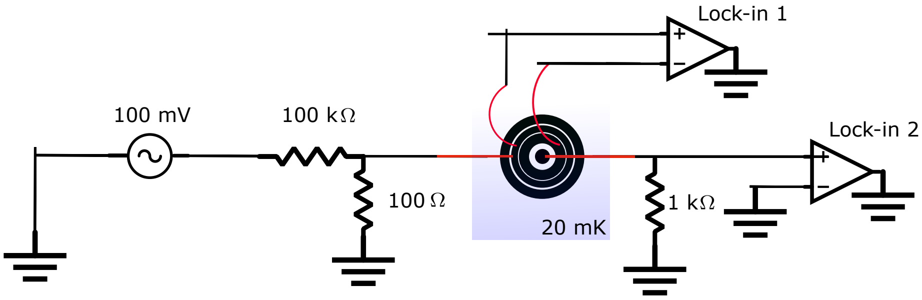

Figure S3: The circuit used to measure the conductance at B$\neq$ 0. This circuit was used in Fig.2 of the main manuscript to compare and verify the result obtained with setup A.

3 Magneto-transport measurement

In Fig.S4 the magneto-conductance measured at low perpendicular magnetic fields with the experimental setup B and is shown. The onset of Shubnikov-de Haas oscillations (SdH) is observed at very low magnetic fields as expected from the very high electron mobility of the 2DEGs. Due to a large magneto-conductance at low magnetic field in a Corbino sample, distinct panels show the conductance over different magnetic field ranges. No evidence was found for a superconducting transition, *i..e* a superconducting critical field, in either samples.

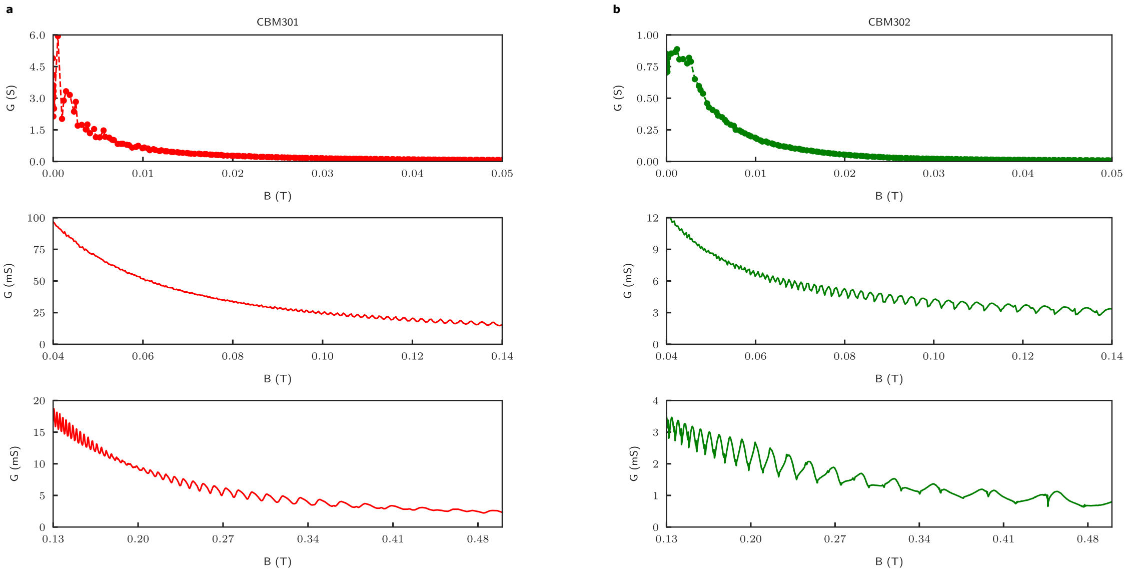

Figure S4: Magneto-conductance measured in (a) CBM301 and (b) CBM302 at 20 mK.

4 Electron density extraction

The SdH oscillations (after reducing the background) of conductivity (or conductance) *versus* inverse magnetic field are given by,

$$\Delta\sigma_{xx} \propto A \;\cos\left(2\pi\left(\frac{B_f}{B} - \delta\right)\right), \tag{1}$$

where the $\delta = 1/2$ and B_f is the SdH frequency. Here, we use the conductivity and conductance terms interchangeably since both parameters are related via a simple geometric factor. Fig.S5 shows the background reduced SdH data and the fit performed for CBM302 at 20 mK. In it, we used the moving average of the raw data which was then subtracted to obtain the background reduced SdH oscillations. The fitting function used Eq 1 to obtain the SdH frequency which was then used to

calculate the electron density n,

$$n = \frac{2eB_f}{h}, \tag{2}$$

where e and h are the electron charge and Planck's constant.

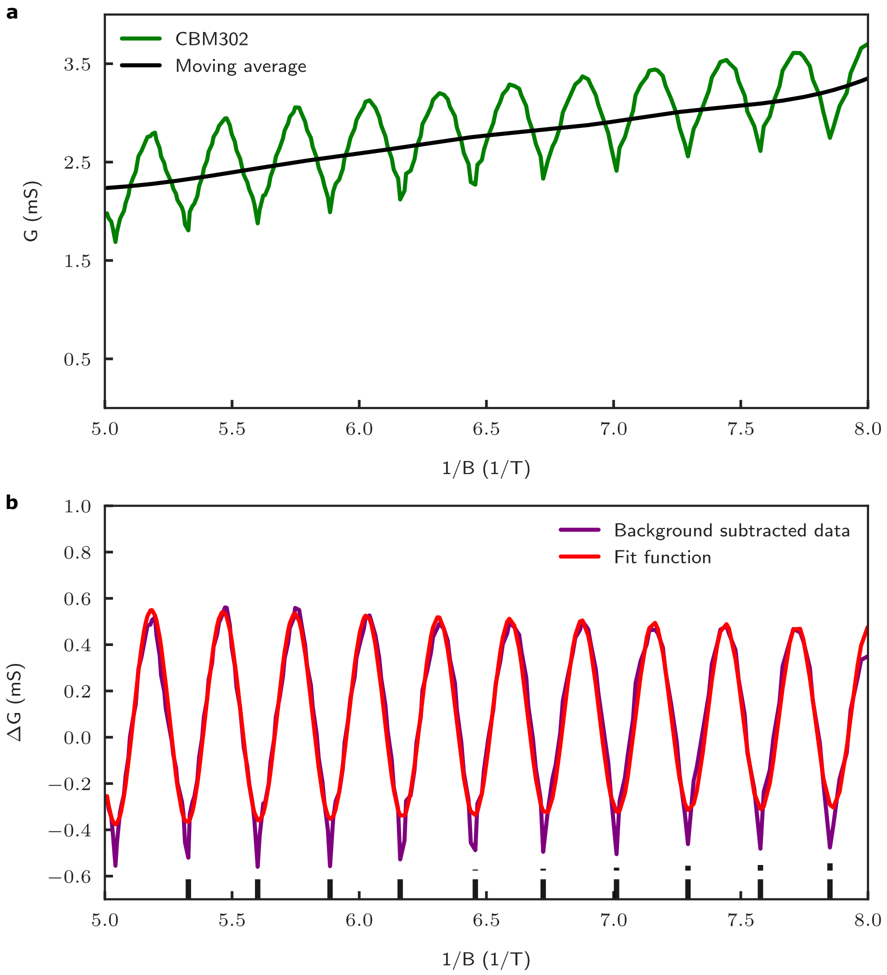

Figure S5: Panel (a) shows the SdH oscillation of CBM302 at 20 mK and the calculated moving average. The background reduced data and fit performed are shown in panel (b).

4.1 Temperature dependence of electron density

The electron density extracted using the aforementioned procedure at different temperatures ranging from 20 mK to 750 mK for CBM301 and CBM302 are shown in Fig.S6. The calculated electron density remains constant within 0.5% in the temperature interval where the anomalous behaviour in electronic transport was observed.

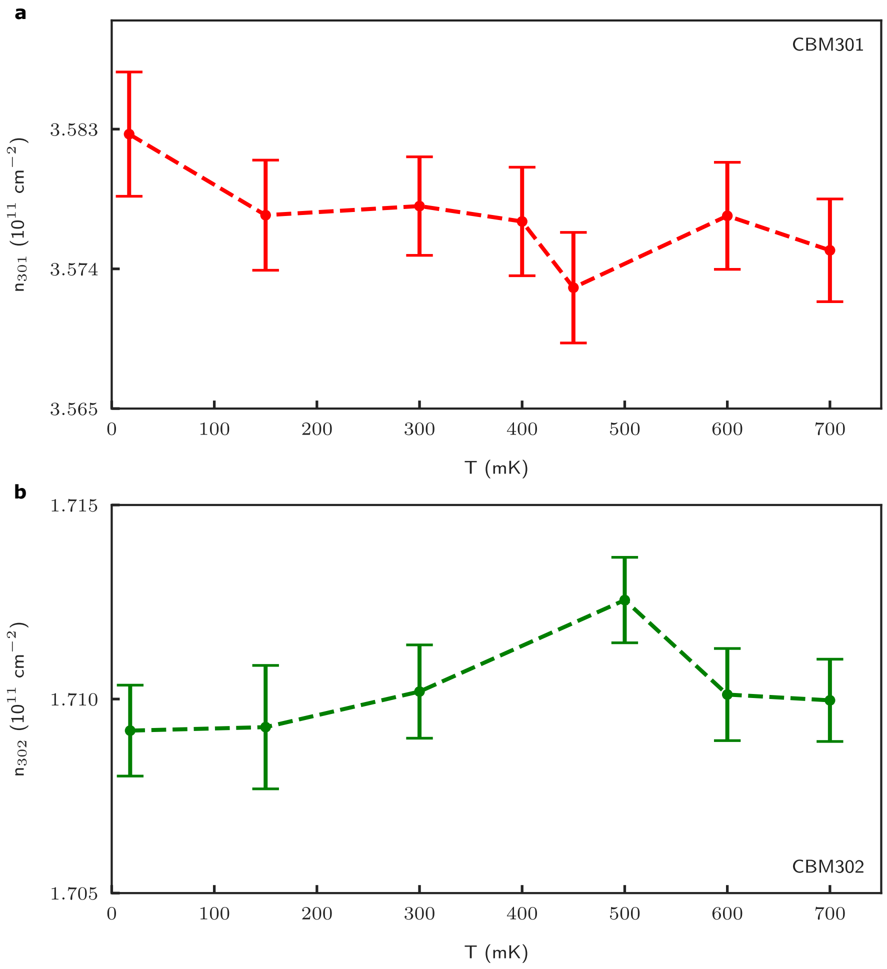

Figure S6: Temperature dependence of electron density for (a) CBM301 and (b) CBM302.

5 Data consistency and reproducibility

The anomalous behavior in resistance observed in CBM301 and CBM302 has been reproduced during several cooldowns. Fig.S7 shows the data obtained for two different cooldowns in the same dilution refrigerator system using experimental setup A for both Corbino samples. In addition to the data shown in Fig.S7, the results have been reproduced several times using both experimental setups in different cooldowns, and also in two different dilution refrigerators with distinctive electronics.

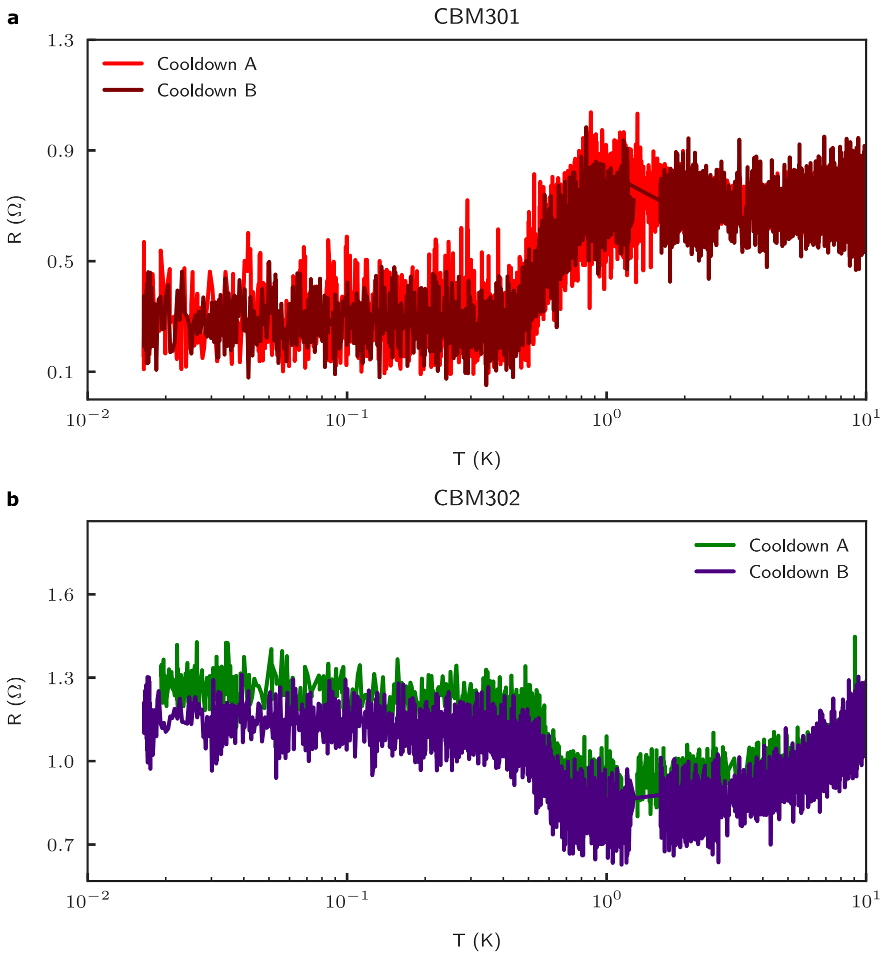

Figure S7: Temperature dependence of resistance for (a) CBM301 (b) and CBM302 measured during two different cooldown.

6 Transport measurements at higher temperature

Electronic transport measurements were also performed at higher temperatures than shown in the main text, and they are shown in Fig.S8. As expected, the monotonic increase in resistance with increasing temperature for VdP301 and VdP302 is observed. Note the increase in resistance observed at ∼400 mK for CBM301 is followed by a nearly-constant resistance value over a wide range of temperature up to ∼10 K, and subsequently a monotonic increase in resistance is observed at higher temperatures.

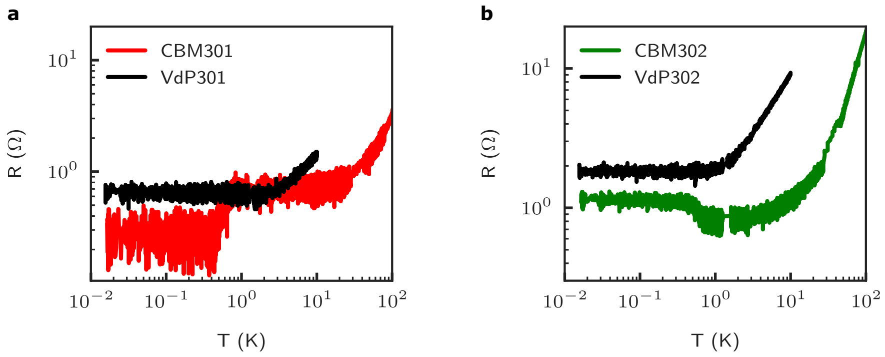

Figure S8: Temperature dependence of resistance up to 100 K for (a) CBM301, VdP301 and (b) CBM302, VdP302.
